# Nocebo effects and participant information leaflets: evaluating information provided on adverse effects in UK clinical trials

**DOI:** 10.1186/s13063-020-04591-w

**Published:** 2020-07-17

**Authors:** Nigel Kirby, Victoria Shepherd, Jeremey Howick, Sophie Betteridge, Kerenza Hood

**Affiliations:** 1 Centre for Trials Research, Cardiff University, Neuadd Meirionnydd, Heath Park, Cardiff, CF14 4YS UK; 2 Nuffield Department of Primary Care Health Sciences, University of Oxford, Radcliffe Primary Care Building, Oxford, OX2 6GG UK; 3 Department of Oncology, University of Oxford, Old Road Campus Research Building Roosevelt Drive, Headington, Oxford, OX3 7DQ UK

**Keywords:** Nocebo, Placebo, Patient information leaflets, Adverse effects, Readability

## Abstract

**Background:**

Nocebo effects (‘negative placebo’ effects) experienced by clinical trial participants can arise from an underlying condition or through communication about side effects in the participant information leaflets (or elsewhere). Misattributing nocebo effects to the medicinal intervention can lead to participants experiencing harmful nocebo effects and may result in distortion of adverse effect reporting. However, little is known about how information on potential side effects is provided to trial participants. There is increasing concern that the way in which potential side effects in clinical trials are described to patients in participant information leaflets (PIL) can in itself cause harm by either increased anxiety, poor adherence or inducing the side effect itself. In this study, we aimed to explore these concerns and identify the way in which potential side effects from investigational medicinal products used in trials are presented in written information to potential participants.

**Methods:**

Trials were identified from the International Standard Randomised Controlled Trials Number (ISRCTN) clinical trial registry (a primary registry of the WHO International Clinical Trials Registry Platform (ICTRP)). Eligible studies were placebo-controlled clinical trials of investigational medicinal products (IMP) in adults conducted in the UK. We assessed readability using the Flesch Reading Ease scale, Gunning-Fog Index and Flesch-Kincaid Grade. Data extracted from the PILs were divided into 8 predefined qualitative themes for analysis in NVivo11.

**Results:**

Most of the patient information leaflets were ranked as ‘fairly difficult to read’ or ‘difficult to read’ according to the Flesch Reading Ease scale. All studies presented information about adverse events, whereas only a third presented information about intervention benefits. Where intervention or study benefits were presented, they were usually after adverse events (21/33, 64%).

**Discussion:**

Participant information leaflets scored poorly on ease of readability and had more content relating to adverse effects than any potential beneficial effects. The way in which adverse events were presented was heterogeneous in terms of their likelihood and severity and the amount and level of detail provided. By comparison, potential benefits from the intervention and/or study were described less often, by shorter text, and only after information about harms.

## Background

The placebo effect is well-documented in the literature and occurs when patients experience an improvement in their symptoms in response to an intervention which is biologically inert with respect to their condition yet which they believe is helpful [1]. The nocebo effect is tantamount to the placebo effect but differs in that nocebo effects arise from a belief that an adverse effect occurs from the patient’s negative expectations [2]. This negative expectation will come from a belief that the drug that they are taking will do them harm. A recent overview of systematic reviews (20 systematic reviews which included 1271 trials across multiple disciplines) found that 50% of trial participants who are allocated to placebo groups experience adverse effects, and 5% drop out of the trial due to ‘drug-induced’ intolerance [3]. The included reviews found that for primary headache disorders the nocebo frequency was 18.67% [4] (56 trials—placebo participants not reported), in RCTs for depression 44.70% (21 trials 3255 placebo participants) [5], in neuropathic pain trials it was 52.0% (12 trials 943 placebo participants) [6], RCTs for pharmacologic treatments for Parkinson’s 64.70% (41 trials 3544 placebo participants) [7], and RCTs for fibromyalgia treatment 67.20% placebo-treated patients reported AEs (16 trials 2016 placebo participants) [8].

However, it is extremely rare [9] for adverse reactions to be directly caused by placebos. There are two overlapping explanations for how this might occur. First, a patient may have an underlying condition whose natural history produces an event (such as a headache), then the patient misattributes the event to the trial intervention (in their case, a placebo). Second, having been warned about side effects in the patient information leaflet (or elsewhere), the patient may expect an adverse effect (AE). This negative expectation could then produce the event [10].

All clinical trials are expected to produce participant information leaflets (PILs) to inform potential participants about the aims of the research, what will happen to them if they decide to take part and what the risks, side effects and benefits are of taking part in the research [11]. Informing patients about any research they participate in is required by the general ethical principle that we must respect patient autonomy and this is clearly stated in the Declaration of Helsinki and the Nuremberg code. More specifically, the PILs are a requirement of Good Clinical Practice (GCP) [12]. There is some evidence, however, that methods for achieving informed consent through the PIL can introduce unnecessary side effects and may therefore violate the ethical requirement to avoid unnecessary harm [13]. In one multicentre randomised trial of aspirin or sulfinpyrazone for treating unstable angina, due to differences in individual hospital review processes, patients either received or did not receive a statement outlining possible gastrointestinal side effects. In two out of the three centres, this resulted in a 6-fold increase (*P* < 0.001) in the number of individuals withdrawing from the study because of subjective, minor gastrointestinal symptoms [14]. This suggests that PILs may prompt patients to expect side effects (nocebo effects), which may cause patients to experience the side effects described.

To date, there has been very little research on how PILs present information about harms. Some recent work exploring participants’ views about information provision has shown that the two most important informational items in a PIL for participants are the possible side effects of trial treatment and the possible disadvantages and risks of taking part in a study. The least important item is whether participants would receive payments for taking part [15]. This shows a clear indication that participants in clinical trials are primarily concerned about the safety information contained in PILs and seek this information out first. The HRA guidance around describing risk and adverse effects in PILs states that ‘A fair and honest evaluation of the consequences of research, including possible significant benefits and harms and their relative likelihoods, must be described to potential participants and that potential participants must be given an honest assessment of the likelihood that something might go wrong, and the consequent level of harm that might be caused’ [11].

In this study, we aimed to increase the evidence base in this area by identifying the way in which potential side effects from investigational medicinal products used in trials are presented in written information to potential participants.

## Methods

### Design

Ongoing and recently completed (within 3 years) trials were identified from the International Standard Randomised Controlled Trials Number (ISRCTN) clinical trial registry https://www.isrctn.com/. Eligible studies were placebo-controlled clinical trials of investigational medicinal products (IMP) in adults conducted in the UK. Three clinical areas were targeted: cancer, musculoskeletal conditions and mental and behavioural disorders. The clinical areas we chose were based on previous evidence that nocebo effects seem likely to affect these areas, namely, musculoskeletal disorders, mental and behavioural disorders and cancer. We chose more than one area in order to compare PILs provided in conditions with contrasting features in terms of treatment aims, types and duration of treatment involved, and anticipated side effects. Eligibility was restricted to either current trials or those within years of completion to reflect current practice.

### Data collection

If the PIL was available on the ISCRTN website, then it was downloaded directly from there. If not, we sent an email to a member of the study team who was listed on the ISCRTN website. An email template was set up asking the named individual for each study to provide the study team with the PIL for the given study. Following sending the original email, the named individual was followed up a maximum of 2 times.

Details of eligible studies were extracted and entered into an Excel spreadsheet. Studies and study documents were allocated a unique reference number and anonymised to remove any identifiable information. Study documents were reviewed for content relating to the intervention and placebo used in the trial. Data was then extracted by three members of the research team (NK, SB, VS) and entered into qualitative data analysis software (NVivo 11).

We used the Flesch Reading Ease Scale, Gunning-Fog Index and Flesch-Kincaid Grade [16] to assess the readability of participant information contained in the PILs. The Flesch Reading Ease Scale and Flesch-Kincaid Grade are able to measure how easily people can understand a piece of text. The Flesch Reading Ease Scale rates the reading ease from 5th Grade to college graduate (The UK equivalent for this is from year 7 to a university graduate). The Flesch-Kincaid Grade rates reading ease using US grade levels via a formula to obtain a reading ease. The Gunning fox index estimates the years of formal education a person needs to understand the text on the first reading; this is given in US school years.

As well as the reading indexes, the data extracted was divided into 8 themes which were predefined before data collection began from the PIL. These are descriptions of the intervention and placebo. Data was also extracted on the adverse effects listed, the likelihood and severity of these events, what to do in the event of these adverse effects taking place and the description of the beneficial effects for both the intervention and the trial itself. The total length of the PIL and length of the text that details the risks and adverse effects was also described.

### Data analysis

We reported descriptive statistics (mean, median, range and interquartile range) to report the word length for the reading scales and the total length of the document for risks and adverse effects and its overall length. This was completed by study type and overall. Readability scores were calculated for each information leaflet in order to assess the accessibility of the information provided to trial participants.

The documents were reviewed in order to ensure familiarity with the text, and the content areas from the study documents were extracted and input into qualitative data analysis software (NVivo 11).

We used the method outlined in Bengtsson et al. [17] to conduct our qualitative analysis. This involved of a series of iterative steps, with the four main stages being decontextualisation of the unit of analysis, recontextualisation, categorisation and compilation. The meaning unit (or coding or content unit) is defined as words, sentences or paragraphs containing aspects related to each other through their content and context.

During the decontextualisation stage, the meaning unit (the words or sentences that are intended to convey an item of information or instruction) were coded, similar to open-coding [18] using a generated coding list. Those identified a priori include any description of a side effect, the likelihood (rarity) of a side effect occurring, the severity of a side effect should it occur, and any advice or instructions to the participant following the occurrence of a side effect. The data was coded iteratively (an approach which is widely used in qualitative content analysis and which allows for changes as new information is discovered), in discussion with the research team, to increase the stability and reliability of the coding process.

During the recontextualisation stage, the meaning units were re-read alongside the original data to ensure the content is adequately captured, with no extraneous ‘dross’ included that is not relevant to the aim of the study [17].

The compilation stage drew on a manifest level of analysis, which remained very close to the original text to describe *what was said* using the visible and obvious [14]. Given the anticipated depth of data contained in these types of documents, a manifest analysis which stays closer to the original meaning and context was appropriate. The data has been summarised narratively according to each theme and category.

## Results

### Descriptive results

The ISCRTN search found 65 studies which were eligible (18 musculoskeletal, 22 Mental and Behaviour disorders and 25 cancer). Only 2 PILs were available on the website so therefore the authors (NK, VS, SB) contacted the study teams to request that they provided their study documentation. Responses were received from 34 studies (52%), these were broken down by 11 musculoskeletal (61%), 13 mental and behaviour disorders (59%) and 10 cancer (40%). All of these studies were included in the final analysis (Table 1).

**Table 1:** Readability scores

Readability Scale	Minimum and maximum	Median	Mean	1st quartile range	3rd quartile range	IQR
Flesch Reading Ease	44.6–70.9	56.3	57	54.3	61.5	7.2
Flesch-Kincaid Grade	7.4–12.4	10.15	10.1	9.225	10.8	1.575
Gunning Fox index	10.3–16.4	12.3	12.4	11.675	13.15	1.475

**Fig. 1:**
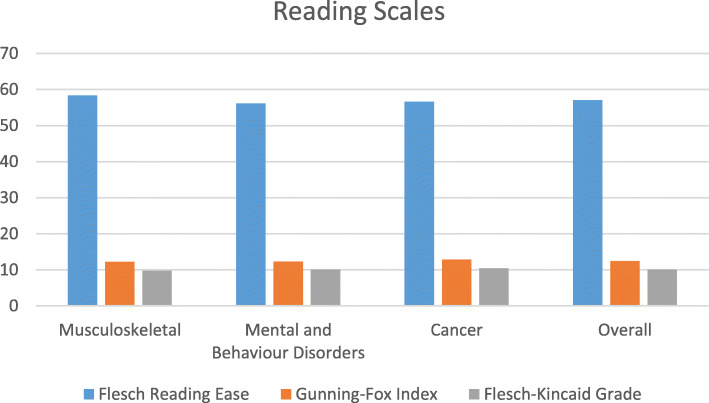
Reading scales by clinical area

The Flesch Reading Ease Scale range across all three clinical areas ranged from fairly easy to read and too difficult to read (Fig. 1). The mean and median scores were interpreted as fairly difficult to read.

The mean reading ease across all clinical areas indicated that all clinical areas were classified as fairly difficult to read (Fig. 1). This indicates that readers overall would need to have a reading age of a 10th–12th grade which in the UK equates to a 16–18 years old.

The Flesch-Kincaid Grade minimum to maximum across all three clinical areas were interpreted as average to skilled reading level. The median and the mean interpretation grades are both at the higher level of average reading ability. This would show that they were not accessible to the general population.

The mean reading grade across all three clinical areas found that all clinical areas had a higher level than the average grade.

The Gunning Fox index range across all three clinical areas were interpreted in the range of high school sophomore (year 11 in the UK) to college senior (2nd year university in the UK). The median score and the mean scores were interpreted as high school senior reading age (year 13 in the UK).

The reading index mean by individual study type are all interpreted as high school senior reading age (year 13 in the UK).

The total length of the cancer PILs was longer than the information leaflet from the other two clinical areas with a mean of 5434 words in comparison to a mean of 2927 in the musculoskeletal studies and 3289 in the mental and behaviour disorder studies.

The length of information leaflets in terms of describing adverse effects and risk in the cancer studies were longer than the other two clinical areas (Table 2). with a mean of 616 words in comparison to a mean of 157 words in the musculoskeletal study and 304 in the mental and behaviour disorder studies. This was also reflected in the percentage of words for adverse effects and risk in comparison to the overall mean.

**Table 2:** Length of documents describing adverse effects and risk

	Minimum and maximum (words)	Median (words)	Mean (words)	% of overall mean (words)	1st QR	3rd QR	IQR
Musculoskeletal	27–236	105	157	5.4	48	291	242
Mental and behaviour disorders	0–529	336	304	9.2	221	364	143
Cancer	127–1165	559	616	11.5	472	643	171
All clinical areas	0–1165	326	354	10.4	127	516	389

Overall, 30 of the trials that were sampled were using licenced medications (9 cancer, 11 mental and behaviour disorders and 10 musculoskeletal); however, only 14 of these (4 cancer, 6 mental and behaviour and 4 musculoskeletal) were using the medication in a way that was used within licenced range of indications, dosage and form.

### Qualitative data analysis

Following the start of the study, the description of the placebo was further divided into sub-themes looking at what the placebo is and why the placebo is being used. We also divided the potential benefits into potential benefits of the study and the potential benefits of the intervention.

#### Description of the intervention under investigation

Reflecting the range of studies included, the data describing the interventions were extremely heterogeneous. The details varied from providing only the drug name and class (e.g. antidepressant (Sertraline)) to extensive descriptions of the intervention and what it is (or usually is) used for, and what the evidence is to date. Interventions in the study documents for cancer trials were generally more extensive than those in the musculoskeletal or mental health groups of studies.

#### Description of ‘placebo’ and the nature of the placebo used

All the included studies involved a placebo intervention as either a substance or a procedure. A small number of the study documents did not provide any information about the placebo used, or what a ‘placebo’ is (5/33, 15%). Where a description was provided of what a placebo is, the majority used the term ‘dummy’ (13/28 46%) or described it as not containing an active ingredient (17/28, 61%), others described it as being an inactive substance (2/28, 7%), or described its physical characterisitics as ‘looking the same’ as the active intervention (11/28, 39%) or not being ‘able to tell which is which’ (1/28, 4%). The effective characteristics of the placebo were described as having ‘no therapeutic properties’ or ‘no anti-cancer action’ (3/28, 11%). Many included a combination of terms, e.g. ‘A placebo is sometimes called a dummy treatment - it looks the same as the actual treatment but does not contain any of the active ingredients and will have no effect on you.’ [ID 204]. Two studies which involved a placebo comparator for a minor surgery intervention described the injection as a ‘sham’ procedure, and another described it as an imitation injection. There were only two study documents which described the ingredients of the placebo, one of which was a saline injection and the other a gelatine capsule.

#### Description of why a placebo is used

The majority of the documents did not provide information about why a placebo was being used (19/33, 58%). Of those that did, a number of different reasons were given. Some described it as ensuring that the patient (6/14, 43%) and/or their doctor (4/14, 29%) would not know whether they had been given the active intervention or not, thus allowing for comparison (3/14, 21%) meaning that the effect of just the active intervention can be measured (4/14, 29%). A small number explained that this reduces bias (3/14, 21%) and means that effects are known to be not just due to chance (1/14, 7%) and makes the trial results more robust (3/14, 21%). Some used a combination of more than one, e.g. ‘If patients are randomised to placebo tablets rather than no active therapy, the doctor and patients will not know which treatment the patients are taking. There is therefore less scope for bias and this makes the results of the trial more robust.’ [ID 103].

#### Description of potential adverse effects

As with the intervention itself, the adverse effects data are heterogeneous in nature, with some study documents containing an extensive bullet-pointed list of in excess of 50 side effects [ID 102], whilst others contained a single short sentence which may include those considered most likely to occur, e.g. ‘The more common side effects are fatigue, insomnia/abnormal dreams and nausea.’ [ID 222] or a brief paragraph [e.g. ID205 and ID 207]. Some studies provided information about how the potential adverse effects are known, e.g. ‘Based on research studies and the experience of other people taking [name of investigational medicinal product], some side-effects can be expected.’ [ID 104]. One study document, for the evaluation of a tea-based intervention, did not contain any information about adverse events [ID 210].

#### Likelihood of adverse effects

‘Almost all of the documents contained information about the likelihood of any adverse effects occurring (30/33, 91%). The three studies which did not provide this information were a medication reduction intervention, a study which involved a single tendon injection, and the tea-based intervention study.’ Of those that did report the likelihood, they were divided between those that used a text description only (16/31, 52%) such as ‘rarely’ or ‘commonly’, and those that combined a probability statement with a text description (15/31, 48%) such as ‘very rarely (< 1 in 1000)’.

#### Severity of adverse effects

Over half of the study documents do not include any information about the severity of potential adverse effects (18/33, 55%). Where the severity of adverse effects are stated, it is described as either ‘mild’ (2/15, 13%); ‘serious’, ‘severe’ or ‘major’ (8/15, 53%); ‘life-threatening’ or ‘fatal’ (2/15, 13%); or a combination of these.

#### Action to be taken by the participant in the event of adverse effects

Many of the study documents (14/33, 42%) did not contain information about what that action the participant should take in the event of an adverse effect occurring. Of those that did, the majority informed the participant that they should contact the study team or doctor or report at the next study visit (12/19, 63%), and/or contact another clinician such as their GP or the nearest A&E department (9/19, 47%). In a small number, the information was contained in a separate information leaflet given to the participant (2/19, 11%), the content of which was not included in this study.

#### Description of the potential beneficial effects of the intervention

A third of the study documents (10/33, 30%) did not refer to any potential benefits from the study intervention. One stated that there would be no benefits for participants receiving the placebo beyond those of the weekly cancer treatment that would be the same outside of the trial [ID 107]. Others expressed hope that the participant would derive some benefit, whilst emphasising that any benefits were unknown or uncertain. Of these, most (11/33, 33%) reported a specific potential benefit such as delaying the time to progression of your disease or helping them to feel stronger or experience a reduction in pain. Four described the potential benefits from the intervention in general or non-specific terms such as ‘the medicine may help you and your cancer’.

#### Description of the potential beneficial effects of the study

The majority of the documents (24/33, 73%) included a statement about the study benifiting future patients or that information from the study will increase understanding about the condition and which treatments are effective, even if the participant does not benefit directly. An additional two stated that some people find it rewarding to take part in medical research. Some of the documents (8/33, 24%) reported that participants may find the additional contact with the research or clinical team reassuring or helpful, or that there may be benefits from the additional monitoring that forms part of the study schedule, or that the study assessments could result in finding a previously unknown condition. Four of the documents stated that there may be benefits from the additional blood tests or scans performed as part of the study. Three documents stated the benefits of receiving an intervention that is not routinely offered by the NHS and so is unavailable outside of the study, or that it was provided free of charge (a vaccination). Two studies described the potential benefits that participants might gain from understanding their condition better through the use of particular study measures. Five explicitly stated that the participant will not benefit from taking part in the study, and three did not include any statement about any potential benefits.

#### Relative position of beneficial and adverse effects

Adverse effects were usually presented before beneficial effects (21/33, 64%). Most commonly, the potential adverse effects and beneficial effects were directly adjacent to each other (19/33, 58%), less often the sections were presented apart by either one page (5/33, 15%) or 2+ pages (4/33, 12%), or dispersed in several locations such as brief details in the introduction with additional information later in the document (2/33, 6%). One document had a detailed description of the potential adverse effects (and the likelihood of each side effect) in a table in an appendix [ID 220], and one had no description of the potential benefits or adverse effects [ID 210].

## Discussion

### Summary of findings

Complex language decreases readability and negatively impacts on the informed consent process [19]. Communication and a lack of clear information play a key role in nocebo effects, and factors relating to information delivery and framing processes can potentially act on a patient’s outcome expectancies that could adversely impact not only treatment effectiveness but also the incidence of side effects [20].

In our sample of PILs, we found that the level of readability is difficult overall. In spite of HRA guidance stating that PILs should be written in non-technical terms that a layperson will understand, most PILs in our analysis required an A level education to understand them.

Our main finding was heterogeneity of the way in which adverse events were presented in PILs. A third of the PILs reported intervention benefits. The style used to present adverse events also varied from single sentences to long bulletpoint lists. However, many PILs are not written from scratch and are often derived from previous information sheets. Other trials may use the HRA template or a Sponsor required template. This is likely to explain the heterogeneity of the presentation.

Informed consent processes may induce nocebo effects, and information framing is known to impact on side effect expectation in clinical practice [21]. There have previously been calls for information about benefits and side effects to be contextualised and for side effect information to be presented in a positively framed way [21]. In this study, adverse events were usually (but not always) presented before benefits and the severity of adverse events was rarely discussed. Whilst the length of the text and the order in which the relevant sections were presented cannot be said to be directly associated with nocebo effects, it will impact on how the information about the potential harms and benefits of the intervention is framed to participants. The heterogeneity and complexity of language in PILs suggest that the framing effects of how the information was being provided were largely unconsidered.

### Strengths and limitations

A strength of this study is that it is the first study that has explored how potential side effects in trials are presented in written information to participants. Another strength is that we have analysed the readability of the PILs in order to explore how readable PILs are overall across multiple disciplines but also, how understandable the adverse effects that are described are to participants.

A limitation of this study is the small numbers of information leaflets that we were able to analyse, and they were drawn from a single database. Initially, 65 studies were considered to be part of this research and only 34 PILs were available for analysis. Whilst this is a limitation of our research, it also highlights that PILs are not as publicly accessible as they potentially could be, especially for studies that are funded by public funders such as the National Institute for Health Research (NIHR). The ISCRTN website contains a section for accessing PILs; however, our research found that it is rare that they are uploaded to the website. We were only able to extract 2 PILS directly from the ISCRTN website.

### Implications for future research

Our analysis suggests that future PILs should follow guidance on readability (generally, by making them more easily understood). Exploring participant’s perceptions of the information provided to them in the PILs is important to explore so see how they are affected by the content and the presentation of the information and the impact of information framing on nocebo effects. Previous research has highlighted the importance of developing participant materials in partnership with patients and other user groups [22]. In addition, feedback from participants who have used these PILs around the nocebo effects would be invaluable to the design of future PILs.

Further research should also focus on exploring how future PILs can be developed which take account of decision-making theories and frameworks which suggest that weighing up the potential risks and benefits of a situation is a key component of decision-making [23].

## Conclusions

Communication of the potential harms and benefits of participating in a trial are important elements of informed consent, and how the relative harms and benefits are framed in participant information materials will impact on participant understanding. Current PILs have low readability and may present information about trial harms that induces nocebo effects. Future research should investigate ways to standardise the information about trial harms so that it is understandable to the lay public and does not induce unnecessary nocebo effects.

## Supplementary information

**Additional file 1.**

## Data Availability

All data are available as a supplementary material.

## Abbreviations

GCP: Good clinical practice
IMP: Investigational medicinal product
ISRCTN: International Standard Randomised Controlled Trials Number
PILs: Participant information leaflets
