# Supplementary material for: Nocebo effects and participant information leaflets: evaluating information provided on adverse effects in UK clinical trials
Source: Trials. 2020 Jul 17;21:658. doi: 10.1186/s13063-020-04591-w (PMC7368797; doi:10.1186/s13063-020-04591-w)
Supplement: Supplementary file 1 — Additional file 1. [file 13063_2020_4591_MOESM1_ESM.docx]

Supplementary Table 1

|  | Minimum and Maximum (Words) | Median  (Words) | Mean  (Words) | 1^st^ QR | 3^rd^ QR | IQR |
| --- | --- | --- | --- | --- | --- | --- |
| Musculoskeletal | 1225-4756 | 2980 | 2927 | 2071 | 3783 | 1712 |
| Mental and Behaviour Disorders | 1209-5133 | 3204 | 3289 | 2778 | 3992 | 1144 |
| Cancer | 2364-9995 | 5636 | 5343 | 4108 | 5764 | 1657 |
| All clinical areas | 1209-9995 | 3359 | 3376 | 2625 | 4861 | 3236 |
